# A Single-Use, Self-Powered, Paper-Based Sensor Patch for Detection of Exercise-Induced Hypoglycemia

**DOI:** 10.3390/mi8090265

**Published:** 2017-08-31

**Authors:** Eunyoung Cho, Maedeh Mohammadifar, Seokheun Choi

**Affiliations:** Bioelectronics & Microsystems Laboratory, Department of Electrical & Computer Engineering, State University of New York-Binghamton, Binghamton, NY 13902, USA; echo16@binghamton.edu (E.C.); mmoham11@binghamton.edu (M.M.)

**Keywords:** sweat sensor patch, self-powered glucose sensor, enzymatic fuel cell, paper-based device, exercise-induced hypoglycemia

## Abstract

We report a paper-based self-powered sensor patch for prevention and management of exercise-induced hypoglycemia. The article describes the fabrication, in vitro, and in vivo characterization of the sensor for glucose monitoring in human sweat. This wearable, non-invasive, single-use biosensor integrates a vertically stacked, paper-based glucose/oxygen enzymatic fuel cell into a standard Band-Aid adhesive patch. The paper-based device attaches directly to skin, wicks sweat by using capillary forces to a reservoir where chemical energy is converted to electrical energy, and monitors glucose without external power and sophisticated readout instruments. The device utilizes (1) a 3-D paper-based fuel cell configuration, (2) an electrically conducting microfluidic reservoir for a high anode surface area and efficient mass transfer, and (3) a direct electron transfer between glucose oxidase and anodes for enhanced electron discharge properties. The developed sensor shows a high linearity of current at 0.02–1.0 mg/mL glucose centration (R^2^ = 0.989) with a high sensitivity of 1.35 µA/mM.

## 1. Introduction

Regular physical exercise benefits patients with diabetes. Physical activity provides effective diabetes management, decreases risk of cardiovascular disease, and improves body composition [1,2]. However, exercise can increase the risk of hypoglycemia in insulin-dependent diabetes mellitus, causing irritability, confusion, and even seizures and unconsciousness [3,4,5]. While endogenous insulin secretion decreases during or after exercise under normal physiologic conditions, diabetics with a loss of insulin secretory capacity can be put in a severe situation because of hypoglycemia, defined as a blood glucose level below 0.7 mg/mL [6]. To reduce hypoglycemic episodes, measurement of glucose levels in blood during and immediately after exercise is critical for patients needing intensified glucose control [3].

Today’s most widespread methods for glucose self-testing involves monitoring the blood glucose levels. Such techniques use a lancet device to manually prick the skin for the blood sample. Then, the sample is positioned onto a disposable test strip, which is inserted into a portable glucometer to read an electrochemical signal and calculate the blood glucose levels [7,8]. However, the conventional measurements are not suitable for preventing hypoglycemia during exercise. This is because (i) the underlying process relies on invasive and inconvenient blood sampling, causing the possibility of sample contamination and skin irritation with sweat containing various electrolytes and proteins, (ii) the method needs patients to carry many accessories during physical activity (e.g., jogging), including lancets, alcohol swabs, and a relatively large glucometer, and (iii) the technique requires a sophisticated electrochemical sensing technique and sufficient electrical energy for applying a constant working potential to the amperometric glucosensor, which makes the technique difficult to be fully integrated in a compact and portable fashion.

**Figure 1:**
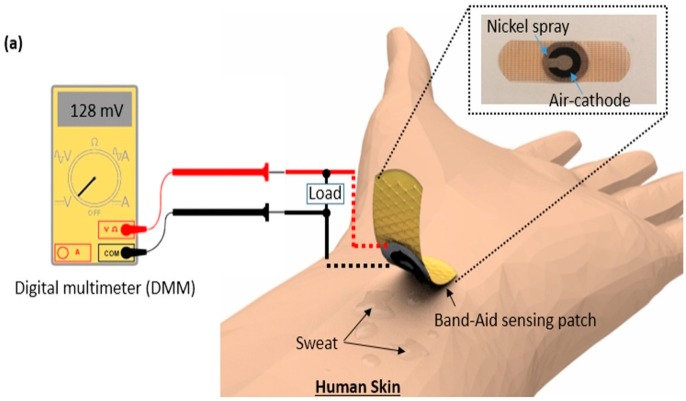

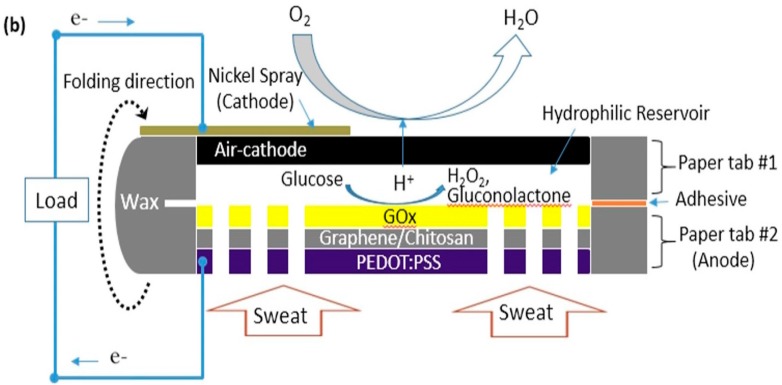
Schematic diagram of (**a**) sweat glucose sensing on skin with our device and (**b**) its cross section. When sweat with different concentrations of glucose is dropped on to the hydrophilic reservoir of the pa-per tab #1, the glucose oxidase (GOx) immobilized on the paper tab #2 catalyzes oxidation reaction of glucose.

In this study, we demonstrate the sensing concept for self-powered, wearable, and disposable glucose monitoring in sweat to detect the exercise-related hypoglycemia (Figure 1a). Sweat has been recognized as an excellent biofluid for non-invasive glucose monitoring, as the glucose level in sweat is metabolically related to blood glucose [9,10,11]. Sweat-based glucose sensing is attractive for managing exercise-induced hypoglycemia because the measurement is performed during or immediately after exercise when there is enough sweat to obtain an adequate sample. This potential alleviates the shortcomings of conventional non-invasive sweat sensors, which can be hampered by the difficulty of collecting enough sweat for analysis, sample evaporation, and the relatively long time required for sample collection [12,13]. Our wearable glucose sensor integrated a vertically stacked paper-based glucose/oxygen enzymatic fuel cell into a standard Band-Aid patch. The paper-based device, attached directly to human skin, wicked sweat by using capillary forces and monitored an electrochemical current generation as a transducing output signal for glucose monitoring in sweat, thus eliminating the requirement of exterior batteries and advanced readout instrumentation. The glucose-sensing device is self-powered because it self-produces a transducing output signal, and the read-out process only requires a simple, widely available and inexpensive digital multimeter (DMM). We will integrate the separate readout instrument into the sensor system in future work. Here, we present a proof-of-concept demonstration of a paper-based enzymatic glucose biofuel cell for the non-invasive monitoring of glucose in sweat. The 3-D glucose/oxygen enzymatic fuel cell was assembled from a 2-D paper sheet by simply folding along a pre-defined crease and attaching it with adhesive spray (Figure 1b). Simply patterning hydrophilic reservoirs with hydrophobic wax and introducing an electrically conducting polymer mixture (poly(3,4-ethylened ioxythiophene):polystyrene sulfonate (PEDOT:PSS)) to the paper made fabrication relatively easy and can be adapted to mass production. In order to overcome the kinetic barrier for enzymatic electron transfer, graphene nanoparticles were introduced to the PEDOT:PSS reservoir. The microporous paper structure provided an efficient mass transfer to the anode and a large surface area, while the PEDOT:PSS polymers and graphene nanoparticles enhanced electrocatalytic reactions and electron transfer rates, resulting in a very sensitive detection of relatively low concentrations of glucose in sweat than in the blood glucose level [13].

## 2. Materials and Methods

**Figure 2:**
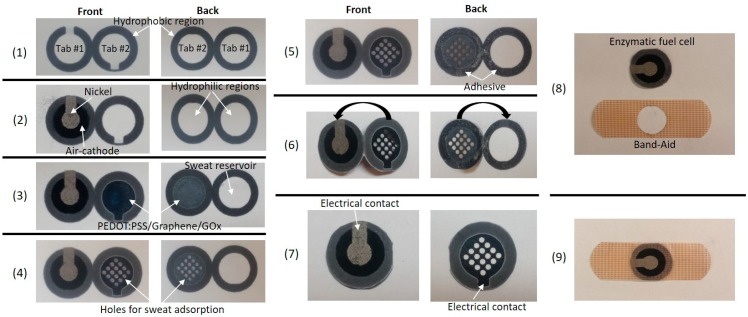
(**1**)–(**9**) Intermediate steps for fabricating the device. Step #(**1**) Form wax-boundary; Step #(**2**) Fabricate air-cathode; Step #(**3**) Form anode and GOx; Step #(**4**) Cut holes; Step #(**5**) Spray adhesive; Step #(**6**) Fold tabs; Step #(**7**) Assemble enzymatic fuel cell; Step #(**8**) Cut hole in the Band-Aid strip; Step #(**9**) Assemble senor patch.

The fuel cell-based sensor was composed of three functional layers; (1) the anodic layer with a conductive reservoir, (2) the sweat reservoir, and (3) the air-cathode layer (Figure 1). The circular-shaped fuel cell was carefully aligned with a hole cut in the Band-Aid patch so that the cathode was exposed to the air for its cathodic electrochemical activity, and the anodic reservoir was directly in contact with human skin (Figure 2).

### 2.1. Reagents

Chitosan (CAS 9012-76-4), glucose oxidase (GOx) from aspergillus niger (CAS 9001-37-0), Nafion^®^ solution (CAS 31175-20-9), D-glucose (CAS 50-99-7), and acetic acid (CAS 64-19-7) were purchased from Sigma-Aldrich (Sigma-Aldrich Corporation, St. Louis, MO, USA). Activated carbon (AC) was purchased from Cabot Corporation (Boston, MA, USA). A conductive nickel spray was purchased from MG Chemicals (Burlington, ON, Canada) for the cathode.

### 2.2. Device Fabrication and Operating Principle

The 2-D sheet of paper (Whatman Grade 1 Qualitative Filter Paper, GE Healthcare, Little Chalfont, UK) was designed to form two bending tabs. Then, we defined the hydrophilic regions by printing wax boundaries with a solid-wax printer (Xerox Phaser 8570dn, Xerox Corporation, Norwalk, CT, USA). Microsoft PowerPoint (version 2010, Microsoft, Syracuse, NY, USA) was utilized to design the wax boundaries on the paper sheet. The front side pattern was printed first and then heat treated at 150 °C for 90 s. The back side pattern was printed after heat treatment of the front side, and was also heat treated at 130 °C for 30 s so that complete penetration of wax from either side can be allowed through the paper. One of the tabs incorporated an air-cathode with a hydrophilic reservoir for sample holding, while the other tab included a conductive PEDOT:PSS treated reservoir for immobilization of GOx (Figure 1b and Figure 2). When a glucose sample was dropped on to the paper reservoir, the GOx immobilized on the anode catalyzed the glucose oxidization. Then, the enzyme performed a direct electron transfer reaction to transfer its electrons to the anode. The electrons flowed to the cathode via the external load. The device determined glucose concentration by monitoring the changes in current output from the enzymatic reactions, developing a self-powered glucose sensor.

### 2.3. Conductive Anodic Reservoir

A mixture of PEDOT:PSS (Sigma-Aldrich) and dimethyl sulfoxide (DMSO, Sigma-Aldrich) in deionized (DI) water was pipetted into the defined anode reservoir to make it conductive and porous, and 3-glycidoxypropy-trimethoxysilane was added to improve the hydrophilicity [14,15]. GOx was immobilized on the conductive reservoir by using a graphene/chitosan solution that had been applied on the anode reservoir before the GOx was introduced. The graphene/chitosan solution was prepared by sonicating 1 mL of 0.5 wt % chitosan solution prepared in 2% acetic acid for 15 min and adding 1 mg of graphene powder (N008P40, Angstron Materials, Dayton, OH, USA). The biocompatible composite of graphene/chitosan was applied as a matrix to immobilize the redox enzyme, GOx. Because chitosan with abundant amino groups provides excellent film-forming ability for enzymes, it is commonly used to disperse nanomaterials and immobilize enzymes for biological sensors [16,17]. GOx solution was prepared by stirring 5 mg GOx dissolved in 0.5 mL 0.1 M pH 7.0 phosphate buffer solution. GOx was used in this work because of its extremely specific catalytic activity towards glucose [18]. The graphene nanoparticles acted as mediator and helped the GOx perform a direct electron transfer reaction to transfer the electrons to the anode [19,20]. The anode with the GOx was air-dried at 4 °C. 16 holes were cut on the anodic layer to provide the inlets for sweat.

### 2.4. Air-Cathode

An activated carbon (AC)-based cathode was fabricated on one side of a paper tab with nickel spray to allow for structural support and to function as a current collector. We previously fabricated the air-cathode on paper by depositing Ni with AC catalyst (15 mg/cm^2^) in a binder solution [21,22]. The catalysts with a binder solution were applied and dried in air for 24 h. The binder solution was mixed with 1200 µL of 5 wt % Nafion solution, 150 µL of DI water, and 600 µL of isopropanol into a conical tube and a mixing well in a vortex machine. Because oxygen is readily accessible, sustainable, and environmentally friendly, the air-cathode based device offers the best promise for this application. Moreover, the proton exchange membrane can be removed from the system to reduce the production cost and increase power generation with better proton travel efficiency through the hydrophilic reservoir.

### 2.5. Measurement Setup

We measured the potentials between the anodes and the cathodes with a data acquisition system (USB-6212, National Instrument, Austin, TX, USA), and recorded the readings via a customized LabView (version 11.01, National Instruments, Austin, TX, USA) interface. An external resistor between the anode and the cathode closed the circuit. The current through this resistor was calculated using Ohm’s law.

## 3. Results and Discussion

Sweat-based wearable glucose sensing has attracted considerable attention because of non-invasive blood sugar monitoring of the wearers [11,23,24]. However, conventional techniques require external power sources and readout instrumentation to transduce output signal for glucose monitoring in sweat, increasing the design complexity and the device cost. Recently, an innovative way to monitor glucose has been proposed that transduces chemical energy into electrical energy by using enzymatic fuel cells [25,26,27,28]. Their electron discharging properties helped to develop self-powered sensing devices, where the GOx at the anode oxidizes glucose in sweat while ACs at the air-cathode reduces oxygen. Therefore, the current output of the glucose enzymatic fuel cell as a transducing signal is corresponding to the concentration of glucose. In early 2016, we reported a paper-based glucose enzymatic biofuel cell for glucose monitoring in human blood and showed its potential as a self-powered glucose sensing device [29]. This was the first application of self-powered glucose monitoring technology for a disposable, inexpensive paper-based device platform. Although the sensor produced enough levels of current and power output to sensitively monitor high glucose concentrations in blood (~1 mg/mL), their capacity will not monitor the significantly lower concentration of glucose in sweat (~0.2 mg/mL). The GOx-based anode screen-printed on paper showed performance limits because of the low efficient electron transfer from the GOx to the electrode surface, and the poor interaction between the enzyme and the electrode. This is because the redox electroactive site of GOx is embedded deeply in the enzyme matrix that hampers the electron transfer between the enzyme and the anode [30,31]. Achieving direct and efficient electron transfer between enzymes and anodes is very important to improve the sensor performance. In addition, we could not harvest all of the electricity that would seem to be available from all of the glucose analytes placed throughout the paper reservoir. Only a small amount of glucose adjacent to the anode in paper contributed to the current generation because of the non-conducting paper matrix cannot be an electron acceptor.

In this work, a graphene/chitosan/PEDOT:PSS composite was tested on the micro-porous paper matrix. The PEDOT:PSS formed the conductive reservoir without blocking the pores of paper, which was still hydrophilic. The Whiteside group proposed this novel method that could co-fabricate electronic and microfluidic structures on paper [14,15]. Their technique found the best-fit solution for our paper-based enzymatic fuel cell applications by providing a fluidic component that allows for the mass transfer of ions and an electrical wire that allows for the conduction of electrons generated from enzymes. This technique enabled a biocompatible, easy-to-fabricate, conductive, and microporous scaffold for the GOx placed in a paper reservoir to efficiently perform a direct electron transfer reaction to the anode. Furthermore, the graphene modification provided nanostructures and activation centers on anodic surfaces to achieve direct electron transfer between the enzymes and anodes [16,19]. In addition, the microporous paper structure provided efficient mass transfer to the anode and a large surface area.

### 3.1. In Vitro Measurement with Artificial Sweat

When the device was assembled, the preliminary in-vitro studies for the sensor were carried out by using various artificial sweat samples (Quantimetrix, Redondo Beach, CA, USA) with different glucose concentrations. The solution wicked through the conductive and hydrophilic reservoirs without any external pumps and electrical power. This capillary adsorption promoted the attachment of the glucose in the sample to the anodic reservoir and an immediate oxidative reaction by transferring electrons from GOx.

Glucose → Gluconolactone + 2H^+^ + 2e^−^ (1)

The large pore sized filter paper (~10 µm) provided a higher volume of sample in the reservoir and ultimately allowed for a longer duration of stable electrical signal production [32]. Oxygen is reduced at the AC catalyst loaded cathode.

O_2_ + 4H^+^ + 4e^−^ → 2H_2_O (2)

The air-cathode part was exposed to the air to maximize the cathodic reduction process. The hydrophilic sweat reservoir was necessary to electrically separate the anode and the cathode. The net reaction in the sensor is shown as:

2Glucose + O_2_ → 2Gluconolactone + H_2_O (3)

**Figure 3:**
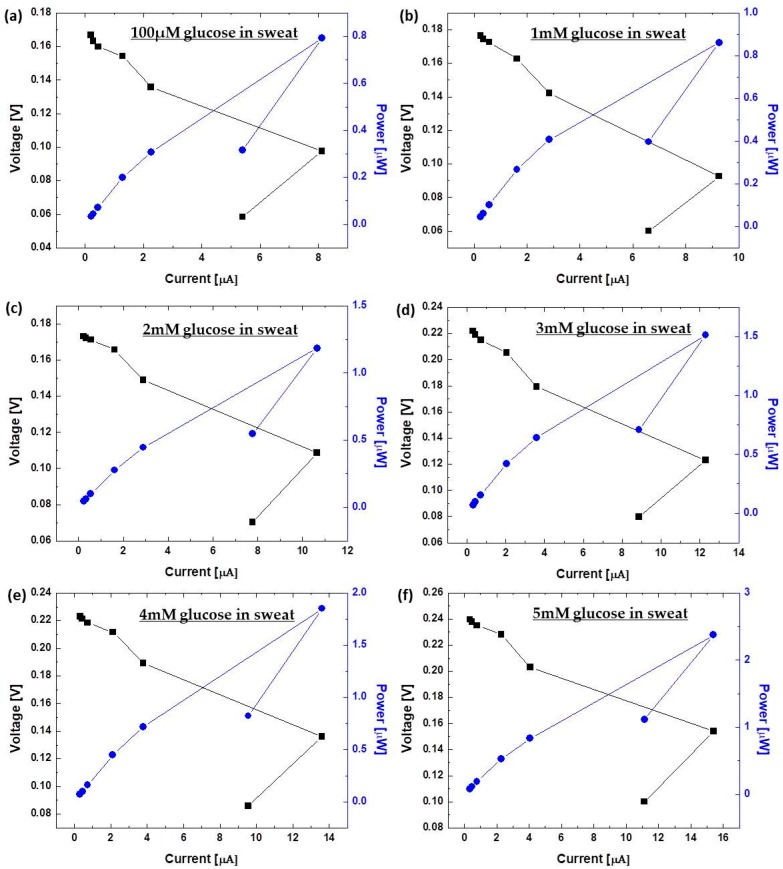
Power outputs and polarization curves with varying concentration of glucose in artificial sweat; (**a**) 0.02 mg/mL (~100 µM); (**b**) 0.2 mg/mL (~1 mM); (**c**) 0.4 mg/mL (~2 mM); (**d**) 0.6 mg/mL (~3 mM); (**e**) 0.8 mg/mL (~4 mM); and, (**f**) 1 mg/mL (~5 mM)). The results are calculated from the maximum current outputs at a given external load (700 kΩ, 500 kΩ, 300 kΩ, 100 kΩ, 50 kΩ, 10 kΩ, and 1 kΩ).

**Figure 4:**
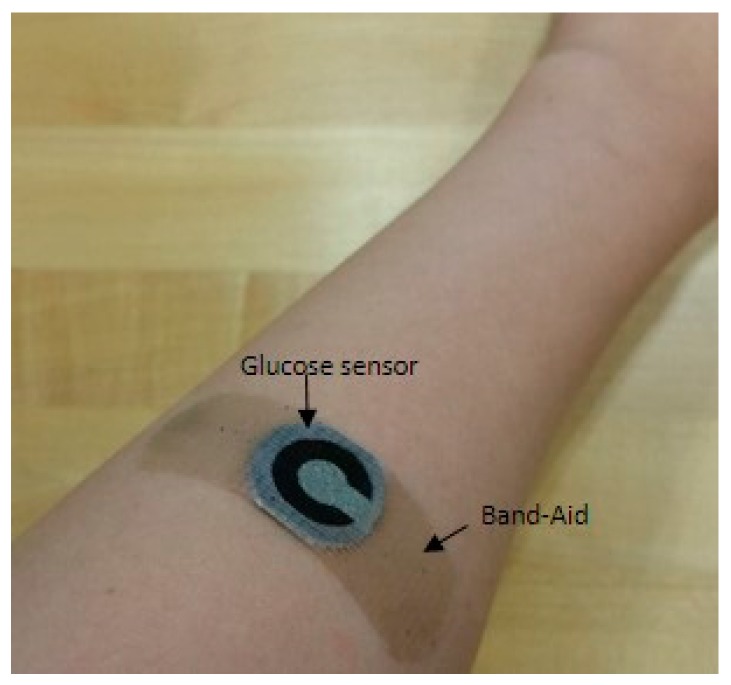
Calibration curve of the sensor (Output current vs. glucose concentration in artificial sweat). The curve of the device with a 10 kΩ resistor shows a high linear range of output current at 0.02–1.0 mg/mL glucose (R^2^ = 0.989) with a high degree of sensitivity of 1.35 µA/mM.

Figure 3 displays power outputs and the polarization curve of the devices with varying concentrations of glucose in artificial sweat (0.02 mg/mL (~100 µM), 0.2 mg/mL (~1 mM), 0.4 mg/mL (~2 mM), 0.6 mg/mL (~3 mM), 0.8 mg/mL (~4 mM), and 1 mg/mL (~5 mM)). The results were calculated from the maximum current output at a given external load (700 kΩ, 500 kΩ, 300 kΩ, 100 kΩ, 50 kΩ, 10 kΩ, and 1 kΩ). From the polarization curve in Figure 3, we evaluated the internal resistances of the fuel cells from the region where the curve demonstrated a near-linear drop (i.e., the ohmic loss region). These values are in good agreement with the external load values where the maximum power density can be obtained from Figure 3 [33]. We obtained the maximum power outputs under a 10 kΩ external load, indicating that the internal resistance of the devices was approximately 10 kΩ, which was significantly lower than that of our recent paper-based enzymatic fuel cell (1 MΩ). The reduced internal resistance obtained in this work is probably because of the enhanced electron transfer efficiency through the graphene nanoparticles, and the enhanced anodic surface area in the 3-D conductive paper reservoir. This paper-based glucose enzymatic fuel cell developed in this work showed notable potential as a self-powered sensing device because it produced enough levels of current and power to sensitively monitor the various concentrations of glucose in the artificial sweat samples (Figure 4). The calibration curve of the device with a 10 kΩ resistor shows a high linearity of output signal at 0.02–1.0 mg/mL glucose (R^2^ = 0.989) with a high degree of sensitivity of 1.35 µA/mM, which is about 70 times greater than our previous sensor [29]. Repeated 6 measurements of each glucose concentration had a relative standard deviation of less than 3.3%. With this work, however, we only focused on the feasibility of our conceptual idea, and set the minimum threshold level on a normal glucose concentration (0.2 mg/mL) in human sweat so that we could monitor hyperglycemic events with the higher glucose concentration. However, for our future work, we will cover all its concentration from 0.02 mg/mL.

### 3.2. In Vivo Measurement with Human Sweat

**Figure 5:**
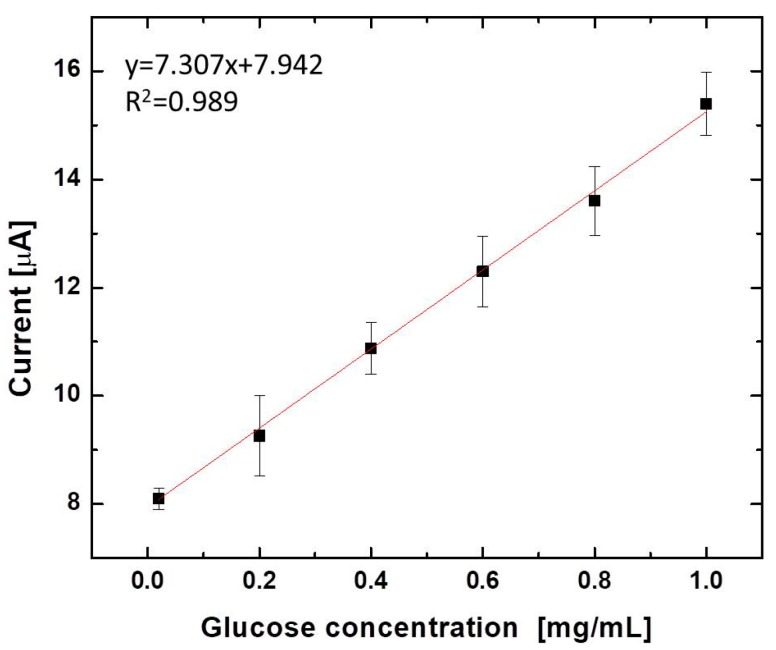
Glucose sensor patch attached to human skin. The glucose levels in sweat were monitored immediately 30 min after the beginning of the exercise.

After demonstrating the in vitro evaluation of the sensors to sensitively measure micro-molar glucose levels, we examined the in vivo measurement of sweat glucose levels in human subjects. The Band-Aid sensor patch was applied to two healthy human subjects (Figure 5), and the glucose levels in sweat were monitored immediately 30 min after the beginning of the exercise (Table 1). We were not able to continuously measure the glucose level from sweat because of many other issues, such as rapid change of the glucose levels during exercise, contamination from the surface of the skin, and the sample degradation. Subject#1 and #2 produced 8.26 µA and 8.10 µA, corresponding to 0.044 mg/mL (244 µM) and 0.021 mg/mL (117 µM), respectively, from the calibration curve in Figure 4. Both are in the normal range of glucose concentration in sweat (<0.2 mg/mL). Also, we compared the sweat glucose with the outputs monitored with a blood glucose meter (OneTouch Ultra Mini Meter Blue, Lifescan, Johnson & Johnson, New Brunswick, NJ, USA). To avoid the time delay issue for sweat glucose versus blood glucose, we immediately measured glucose levels from blood 30 min after the beginning of the exercise. The results indicate a strong correlation between sweat glucose and blood glucose [11,34,35]. Normal blood glucose levels range between 0.9 mg/mL and 1.4 mg/mL [13]. While substantial recent activities implement wearable sensors for real-time, non-invasive health monitoring, this simple sensing platform will add new insight to this field [10,36,37].

## 4. Conclusions

In this work, we reported a paper-based, self-powered sensor patch that allowed for the non-invasive monitoring of glucose in human sweat. This new sensor utilized (1) a screen-printed graphene/chitosan/GOx anode, (2) an air-cathode, and (3) a PEDOT:PSS treated conductive anode reservoir for higher device performance and practical applications. The device was based on glucose/oxygen enzymatic fuel cells using an electrochemical current generation as a transducing signal for glucose monitoring in sweat, establishing a self-powered, low-cost, and disposable glucose sensing platform for preventing exercise-related hypoglycemia. Furthermore, the sensor bandage patch allowed for intimate contact with human skin. Although the sensing system was not fully integrated into the paper and a detector was still needed for glucose monitoring, the equipment was simple and inexpensive. The sensing platform holds considerable promise for efficient diabetes management, and a fully integrated system with a simple readout can be realized toward continuous non-invasive glucose monitoring.

## Figures and Tables

**Table 1:** Glucose levels of human sweat and blood.

In Vivo Measurement	Blood	Sweat	
	Glucose Level Measured from a Glucose Meter	Current at 10 kΩ	Glucose Level Estimation from the Calibration Curve
Subject #1	0.96 mg/mL	8.26 µA	0.044 mg/mL
Subject #2	0.85 mg/mL	8.10 µA	0.021 mg/mL
